# Standardized Outcomes for Randomized Controlled Trials Targeting Early Interventions in Patients With Moderate-to-Severe Traumatic Brain Injury: Protocol for the Development of a Core Outcome Set

**DOI:** 10.2196/54525

**Published:** 2025-01-09

**Authors:** Raphael Cinotti, Yvan Derouin, Amandine Chenet, Lydia Oujamaa, Bertrand Glize, Yoann Launey, Claire Dahyot-Fizelier, Emmanuelle Cartron, Melodie Renvoise, Benedicte Sautenet, Veronique Sebille

**Affiliations:** 1 Department of Anaesthesia and Critical Care Centre Hospitalier Universitaire de Nantes Nantes France; 2 INSERM, Methods in Patient-Centered Outcomes and Health Research, SPHERE, F-44000 Nantes Université, University of Tours Nantes France; 3 Department of Rehabilitation Hôpital Saint-Jacques Centre Hospitalier Universitaire de Nantes Nantes France; 4 SRPR 42, Groupement de coopération sanitaire Centre Hospitalier Universitaire de Saint-Étienne Saint-Etienne France; 5 GIN U1216 Grenoble Institute of Neurosciences La Tronche France; 6 Service de Médecine Physique et Réadaptation Pôle de Beurosciences Cliniques Centre Hospitalier Universitaire de Bordeaux Bordeaux France; 7 HACS team-U1219 Institut National de la Santé et de la Recherche Médicale Bordeaux Population Health & University of Bordeaux Bordeaux France; 8 Anesthesia and Intensive Care Unit Centre Hospitalier Universitaire de Rennes Rennes France; 9 Intensive Care and Anesthesia Department Centre Hospitalier Universitaire de Poitiers Poitiers France; 10 INSERM U1070, PHAR2 Université de Poitiers Poitiers France; 11 Département Universitaire des Sciences Infirmières Épidémiologie Clinique, Évaluation Économique Appliquées aux Populations Vulnérables Paris France; 12 Centre Nantais de Sociologie Nantes Université Nantes France; 13 Service de Néphrologie-Hypertension Artérielle, Dialyses, Transplantation Rénale Centre Hospitalier Universitaire de Tours Tours France

**Keywords:** core outcome set, outcomes research, patient-centered outcomes, traumatic brain injury, patient outcome, head trauma, patient-centered

## Abstract

**Background:**

With more than 60 million new cases around the world each year, traumatic brain injury (TBI) causes substantial mortality and morbidity. Managing TBI is a major human, social, and economic concern. In the last 20 years, there has been an increase in clinical trials in neurocritical care, leading mostly to negative results. The evaluation of neurological outcomes, predominantly as primary outcomes, using clinical scales (Glasgow Outcome Scale) has limitations that could explain these results. Moreover, patient-centered outcomes are seldom reported despite their recognized clinical relevance.

**Objective:**

The aim of this project is to establish a core outcome set (COS) for patients with moderate-to-severe TBI in randomized control trials in neurocritical care research.

**Methods:**

This study will follow five distinct steps: (1) systematic review to identify outcomes that have been reported in trials; (2) semistructured interviews with patients and their families to identify their priorities after TBI and explore potential patient-centered outcomes; (3) health care stakeholder focus groups with clinicians, researchers, and policy makers to describe potential outcomes; (4) an eDelphi survey with stakeholder groups to make a list of previously identified core outcomes; and (5) a consensus workshop to establish a COS for moderate-to-severe TBI clinical trials.

**Results:**

The systematic review was published in August 2024. Regarding Step 2, 30 semistructured interviews of patients and relatives were performed from July 2021 to December 2023, and analyses were completed in October 2024. Step 3 is currently under development, and Step 4 is planned for the end of 2025. Step 5 is expected to occur during fall/winter 2026.

**Conclusions:**

Establishing a COS, to be consistently measured and reported in TBI trials in neurocritical care will ensure rigorous reporting, avoid bias, and improve the integrity, transparency, and usability of clinical research. The French context of the study is the main limitation, but we are seeking international collaboration on the project. The results of each step of the project will be disseminated through abstracts, publications, and patient associations.

**International Registered Report Identifier (IRRID):**

DERR1-10.2196/54525

## Introduction

Despite advances in critical care medicine in the last decades, mortality in patients with traumatic brain injury (TBI) remains high at approximately 15% after trauma [1]. Survivors of TBI experience major consequences: mood disturbances [2], memory loss, neuropsychological impairment [3,4]. Caregivers also experience major burdens with strain, isolation, and disappointment [5,6].

Many work groups recommend that various outcome domains or outcome measurements [7,8] be collected to assess neurological recovery after TBI, mainly in the continuum of rehabilitation. Most often, this involves a combination of multiple measurement scales that collectively capture all dimensions affected by a TBI [9,10].

Nevertheless, in neurocritical care, the primary outcome in most randomized controlled trials (RCTs) is the Glasgow Outcome Scale [1,11]. Indeed, given the broad spectrum of sequelae after acute brain injury, Jennett and Bond [12] proposed the use of the Glasgow Outcome Scale in 1975 to assess neurological recovery after TBI. Since then, this 5-grade scale (death, vegetative state, severe recovery, moderate recovery, and good recovery) has been extensively reported in the neurocritical care literature to compare the efficacy of various treatments after trauma [13-15]. The Glasgow Outcome Scale was considered too simplistic, leading to the creation of an 8-grade extended version in 1981 [16]. The Extended Glasgow Outcome Scale is used extensively in neurocritical care literature to evaluate neurological recovery, often as a primary outcome in clinical trials [17].

Recently, major concerns have been raised regarding the relevance of these scales and their methodological limitations in neurocritical care clinical research. The Glasgow Outcome Scale has substantial interobserver variability, albeit possibly decreasing over time [18]. Nevertheless, this puts score reliability and accurate patient classification into question [19,20], which could lead to erroneous results despite rigorous analysis. Moreover, analysis of data from this scale is often inappropriate, since these data are generally considered continuous when they are ordinal [21]. In addition, assessment conditions can vary and affect patient evaluation. Evaluation can be performed via telephone [22] or face-to-face interviews, for only brain injury or all injuries (brain and peripheral body parts), or by trained or untrained assessors. These variables are frequently not described in other studies [3,11,18]. However, it is recommended that such assessments should be performed by a certified expert in the presence of the patient and caregiver [23]. Certain authors have recently questioned the consistency between these scales and patient quality of life [24,25]. This raises the question of a patient’s understanding of their situation and handicap, which is never evaluated in randomized controlled trials, despite being recognized as an important factor [26]. The aforementioned methodological issues and limitations associated with these scales could explain the litany of negative results in neurocritical care research over the past 30 years [1,15,17,27,28].

Core outcome sets (COSs) can minimize reporting bias, promote consistency in clinical trials, enable direct comparisons of the effect of different interventions, and ensure that outcomes are relevant and important to patients, health care professionals, and caregivers [29]. Hence, the use of COSs for patients with TBI in neurocritical care could reduce the considerable inefficiencies in biomedical research. With more than 60 million new cases of TBI diagnosed globally each year [30,31], there is an urgent need to develop COSs in this area. Accordingly, this project aims to establish a COS for trials in patients with moderate-to-severe TBI included in interventional trials performed during the early phase of the pathology and other types of research including observational studies (eg, registries and other quality indicators for clinical care). This core outcomes set will improve the quality of trials, respond to patient needs, and help rectify decades of negative results.

## Methods

### Overview

The project will be developed in 5 steps according to the methodology of the Core Outcome Measures in Effectiveness Trials (COMET) initiative. The aim of the COMET initiative is to create a COS built by stakeholders, including patients [32,33]. First, we will perform a systematic review of the outcomes in TBI clinical research at the acute phase. Second, we will organize semistructured interviews with patients who survived moderate-to-severe acute TBI and their family caregivers. Third, we will organize semistructured interviews and focus groups of stakeholders involved in the health care pathway of patients with TBI. Fourth, we will perform an eDelphi survey. Finally, a consensus workshop will finalize the process.

### Step 1: Systematic Review

Step 1 was completed in August 2024, and the original article was published [34]. We identified 29 domains related to the 557 different outcomes that will be used for interviews with health care professionals (Step 3) and the eDelphi survey (Step 4).

### Step 2: Semistructured Interviews With Patients and Their Families

#### Explanation and Overview

We will organize semistructured interviews with patients in the late phase of TBI and their family caregivers. To the best of our knowledge, semistructured interviews involving both patients with TBI and their families have been poorly explored. Throughout the qualitative research, we will follow the Interpretative Phenomenological Analysis (IPA), a methodology described by Smith et al [35].

#### Participants and Recruitment

Patients with TBI aged 18 years and older and a family caregiver (the patient's choice) will be eligible to participate. A purposive sampling strategy will be used to organize a minimum of 15 semistructured interviews with the dyad (patient and their family caregiver). The aim is to perform individual semistructured interviews with patients alone and then perform another individual dedicated interview with relatives alone to avoid cross-contamination of interview responses between the 2 parties. However, given the potential challenges for patients (mood disorders, speech disorders, and difficulties in concentration), relatives may attend the patient’s semistructured interview to assist them. The final interviews will depend on when data saturation is reached. We will define saturation as the point when new themes or variations of a given theme cannot be identified. Participants will be recruited from participating centers across France (Bordeaux, Nantes, Rennes, and Saint-Etienne, along with any other contacted centers). The patients and their caregivers will be asked by physical and rehabilitation specialists to participate in the study. Patients in the late phase of TBI will be recruited—defined as the moment when a patient has been discharged at least partially, meaning they no longer require full-time care in a health care facility. The consequences of TBI on their social and personal environments will be assessed.

The steering committee will carry out a sampling strategy to achieve maximum variation in demographics (age, sex, and socioeconomic status) and clinical characteristics (patients with good or poor recovery). We will follow the Consolidated Criteria for Reporting Qualitative Health Research (COREQ) guidelines [36].

#### Data Collection

Each semistructured interview, performed with an IPA approach according to participants’ life experiences, will last a maximum of 90 minutes and take place remotely via videoconference. This is due to the uncertainties in the aftermath of the COVID-19 pandemic. In any case, these interviews will take place outside the hospital to encourage open discussion and limit the stories due to the feeling of disempowerment that could occur in a clinical setting. Each interview, performed by a nurse researcher (senior in qualitative research) and a PhD student, will cover the following for both patients and families: (1) an introduction (5 minutes), where the facilitator will explain the aims of the study and ask the participants to introduce themselves; and (2) the interview (60 minutes), where the participants and families will be asked to discuss their experiences of living with the consequences of TBI, including perceived benefits, harms, and complications related to the pathology and its treatments. The topic guide was tested with 3 dyads and adjusted accordingly (Multimedia Appendices 1-2).

#### Data Analysis

The interview transcripts will be imported into NVivo 12 software (v.1.8; QSR International) [37] to facilitate qualitative data analysis. We will follow the 7 IPA steps described by Smith et al [35]: (1) reading and rereading, (2) exploratory noting, (3) constructing experiential statements, (4) searching for connections in experiential statements, (5) naming the personal experiential themes and consolidating and organizing them, (6) moving to the next case, and (7) working with personal experiential themes to develop group experiential themes across cases. The IPA method reflects the experience, beliefs, values, attitudes, and reasons underlining participant choices, along with those of their relatives. The preliminary themes will be discussed with other investigators to ensure that the full range and depth of data are captured (investigator triangulation) and with patient associations for the full themes.

### Step 3: Stakeholder Interviews

#### Explanation and Overview

Field investigations, in-depth and semistructured interviews, and focus groups will be performed to capture the expectations and practices regarding the management of TBI patients and to detail the range and depth of individual values, beliefs, and attitudes toward outcomes. These interviews or focus groups will not quantify the frequency of opinion. Reporting will adhere to the COREQ guidelines.

#### Participants and Recruitment

Interviews and focus group discussions will be conducted with the following stakeholder groups: (1) health care providers (anesthesiologists, intensivists, neurosurgeons, rehabilitation specialists, nurses, and psychologists); (2) representatives from research, funding, policy, and other stakeholder organizations; and (3) patients and their relatives. A minimum of 60 stakeholders is expected at this point and will be identified from the investigator networks and by snowball effect. At this stage, the steering committee will reach out to stakeholders in other countries to participate in specifically dedicated interviews. Participants will be identified to obtain a maximum variation of representation in professional experience and responsibilities (health care providers and representatives from stakeholder organizations). Recruitment will continue until saturation has been achieved. Informed consent will be obtained from all participants and specific institution review board (IRB) approval will be sought.

#### Data Collection

From a grounded theory perspective [38], field investigations will be conducted in intensive care and rehabilitation units. Interviews and focus groups will incorporate the results from the systematic review and semistructured interviews with patients and their families, allowing other stakeholders to discuss these elements as well. Stakeholders will be asked to reflect and talk about (1) caring for patients with TBI, (2) the benefits and harms of TBI-related outcomes, (3) outcomes believed to be relevant and important to include in future clinical research, and (4) the results obtained from the semistructured interviews with the patients and their families. Face-to-face interviews will be conducted; however, if this is not feasible, web or telephone conferences will be arranged. Each interview will last between 60 and 120 minutes. The interviews will be recorded and transcribed verbatim.

A field researcher has been hired for a 24-month postdoctoral position to complete Step 3. She is a former intensive care nurse with a PhD in sociology from the University of Nantes and has extensive experience in field investigations and conducting interviews. She has no conflicts of interest related to the topic.

#### Data Analysis

Data analysis is expected to begin in 2025. Verbatim transcripts from interviews and focus groups will be transcribed word for word and anonymized. Following the grounded theory methodology [38], each transcript will undergo a constant comparison across individuals and stakeholder groups. Analytical themes will be developed inductively to identify the concepts relevant to the participants, from which a list of outcomes will emerge.

### Step 4: Delphi Consensus Survey

#### Explanation and Overview

At this stage, international contacts will be established to conduct an international eDelphi survey [29,39,40]. This survey will gather opinions and organize the outcomes into a prioritized list. The Delphi method is an iterative consensus technique involving sequential surveys completed anonymously by a panel of participants with relevant knowledge and expertise, ensuring equal influence among all participants [41]. We will aim to retain a minimum response rate of 70% for all rounds.

#### Participants and Recruitment

There is no standard sample size required for Delphi processes. At this point of the protocol, there is no goal regarding the minimum number of stakeholders that will be involved in the Delphi process (ie, patients, family caregivers, nurses, allied health professionals, policy makers, and clinicians in critical care, rehabilitation, and neurosurgery). To ensure maximum variation in sampling, participants will be recruited by using a similar strategy, and approximately one-third of each stakeholder group will be recruited from the participating regions. The participants will be recruited through participating hospitals or institutions and patient organizations. Informed consent will be obtained from all participants.

#### Data Collection

##### Overview

The list of outcomes will be obtained from Steps 1, 2, and 3. The outcomes will be listed individually and grouped under each relevant domain according to the COMET initiative definitions [32,42]. The survey will be reviewed by the steering committee. The surveys will be completed over the internet by using a unique identifier, which will enable us to identify participants completing all 3 rounds of the eDelphi survey. At least 3 reminders will be sent to participants during the Delphi rounds.

##### Round 1

Participants will be asked to rate each outcome using the Grading of Recommendations, Assessment, Development, and Evaluation (GRADE) process [43]. The process recommends a 9-point Likert scale to rank importance. Rankings between 7 and 9 indicate outcomes of critical importance, those between 4 and 6 indicate outcomes that are important but not critical, and those between 1 and 3 indicate outcomes of limited importance. An option “unable to score” will also be available. All outcome domains will be randomized to minimize ordering bias. Participants can suggest additional outcomes and provide reasons for their rankings. The additional outcomes will be recorded (if not duplicated with a previous outcome), grouped in the relevant outcome domain by 2 members, and reviewed by the steering committee. We will review the distribution of scores for all outcomes for each stakeholder group (ie, patients/caregivers, clinicians, health professionals, etc). Any outcomes with a median or mean over 7 will be retained for round 2, along with additional outcomes retained by the steering committee.

##### Round 2

Participants will review the group scores and their own scores for each outcome. They will rerank the outcomes (including additional outcomes identified in round 1) using the 9-point scale and explain the reasons for any changes in their scoring. An outcome with a median or mean over 7, and with 70% or more participants in both stakeholder groups (ie, patient/family member and health professionals) rating the outcome to be of critical importance (7-9), will be included in round 3.

##### Round 3

Participants will be shown their own scores, along with the distribution of scores for each outcome across all stakeholder groups and within individual stakeholder groups. A summary of the results from Steps 2 and 3 will be provided. Participants will be asked to rerank all outcomes and indicate whether they should be included in the COS. To assess the relative importance of the outcomes, they will choose the most important and least important in each outcome domain.

At this point, the recruitment of stakeholders for Step 4 and the Delphi process will not be undertaken for at least 2 years. Data analysis of Step 4 (Delphi process) will be further elaborated and will not be more detailed at this stage.

### Step 5: Consensus Workshop

A face-to-face consensus conference will be held for stakeholders to review, comment on, and endorse the COS. This conference will be chaired by members of the steering committee. As of yet, the number and origins of participants at the workshop have not been established. This sample size is based on the Outcome Measures in Rheumatology (OMERACT) consensus workshop. Purposive sampling will be carried out to ensure maximum variation of demographic and clinical characteristics. Informed consent will be obtained from all participants. All discussions will be recorded and transcribed. The overall conference program is outlined in Textbox 1.

**Figure 1:**
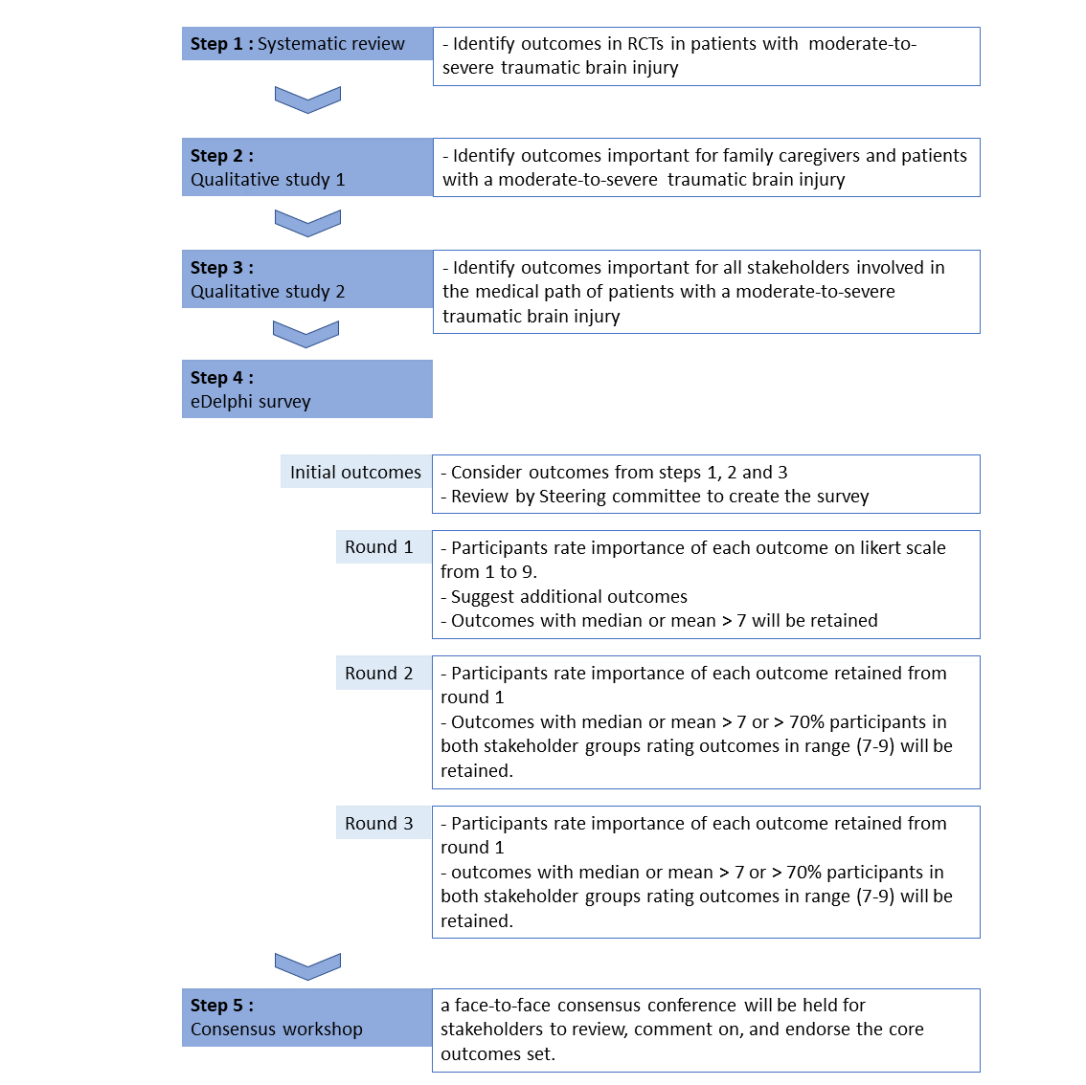
Summary of the project regarding the development of a core outcome set in neurocritical care for patients with traumatic brain injury (TBI). RCT: randomized controlled trial.

Given the timeline of the project, Step 5 will not be undertaken for another 3 years (around mid-2026). The articulation, organization of the workshop, and data analysis will be further elaborated but not detailed here. Figure 1 articulates the differences between the 5 steps.

**Conference program details.**

1. Presentation of results: Detailed results from Steps 2, 3, and 4 will be distributed to the participants. The results will be presented during a plenary session of the consensus workshop, and the outcomes will also be shown according to the consensus classification.
2. Breakout group discussion: Participants will be divided into several groups. A trained facilitator will moderate a group discussion on the results from Steps 3 and 4, consensus classification of outcomes, similarities and differences in stakeholder groups, and the resolution of any disagreement, uncertainties, or issues identified.
3. Plenary discussion: Each breakout group will present a summary of their discussion. The conference chair will moderate the discussion.
4. Endorsement of core outcome set (COS): Participants will be asked to formally endorse the core outcomes set which will include the outcome classified as consensus.

### Ethical Considerations

All steps will be performed according to the appropriate guidelines and regulatory processes.

### Step 1

Ethical approval is not required for this step.

### Step 2

Patients and relatives provided informed consent to participate in the semistructured interviews (CERAR IRB 00010254 - 2022–093). The analysis of the interview transcripts will be anonymized to respect privacy and anonymity. Finally, patients and relatives will receive €50 (US $52.12) each for compensation for their time to perform these interviews.

For the elaboration and recruitment of patients for Step 2 (semistructured interviews), the group is currently working with the association of patients and families (“Union Nationale des Associations des Familles de Traumatisés Crâniens”).

### Step 3

Interviews and focus groups with health stakeholders will also comply with rules and regulations. Although no IRB is necessary in France regarding surveys [44], oral consent will be collected from participants. Verbatim analyses and data will be anonymized and deidentified.

### Step 4

No health data are collected in Step 4. Moreover, IRB approval is not mandatory in France for this type of research. However, we will comply with all national authorities’ guidelines in case of international collaboration. We will also comply with laws and regulations for the publication process.

### Step 5

No health data are collected in Step 5. Moreover, IRB approval is not mandatory in France for this type of research, but we will comply with all national authorities’ guidelines in case of international collaboration. We will also comply with laws and regulations for the publication process.

## Results

Step 1 was completed, and the paper was published in August 2024 [34]. This systematic review was performed from January 2021 to October 2023.

Regarding Step 2, 30 semistructured interviews were carried out (15 patients and 15 caregivers) throughout France. The interviews were performed between July 2021 and December 2023. The results of the verbatim analysis (ie, group experiential themes through cases) were finalized in September 2024, and we expect to begin the publication process by the end of 2024. We aimed to understand the quality of life of both patients and family caregivers, focusing on what matters most to them in their daily lives. The elaboration of Step 3 began in May 2024. The aim of this part of the project is to understand different stakeholder points of view regarding the choice of end points in clinical research.

Although Steps 2 and 3 have not yet been achieved, we have already begun engaging with the scientific community, clinicians, health professionals, other health care stakeholders, and patient associations for the upcoming Delphi process, which is scheduled for the beginning of 2026. This proactive approach ensures that we will have the most comprehensive group of participants for the Delphi process. Step 5 will mark the finalization and dissemination of the COS. This step is preplanned to start in fall/winter 2026.

## Discussion

### Principal Findings

This project introduces a novel clinical research methodology for TBI through the development of a COS. This COS includes a rigorous multidimensional evaluation of the outcome of patients and encourages the integration of Patient-Reported Outcome Measures (PROMs) in this context. We believe this initiative will address decades of negative findings on this topic, emphasizing the urgent need to improve patient outcomes amid strained resources. The project involves various stakeholders, especially patients and family caregivers, in a rigorous methodological approach that has been successfully tested in other medical disciplines [32,45].

Many work groups have published common data elements [46,47] recommended for RCTs. However, these common data elements are unsuitable for RCTs targeting early interventions in neurocritical care. Notably, our systematic review [34] highlighted the low use of patient-reported outcomes in RCTs, despite increasing advocacy for their use by researchers [26,45].

The study's French context is its main limitation, but we are pursuing international collaboration to ensure its global relevance. Furthermore, given that health systems in high-income countries are often comparable, the findings should be globally applicable.

The results of each step will undergo scientific validation and publication. The dissemination plan includes communications in both national and international congresses involving key stakeholders in TBI research, such as anesthesiologists, intensivists, neurosurgeons, trauma leaders, neuropsychologists, and nurses. Targeted congresses include the French Society of Anesthesia and Intensive Care, European Society of Intensive Care Medicine, the European Society of Anesthesia and Intensive Care, and the International Brain Injury Association—among others. Publications will be submitted to peer-reviewed journals. Where resources allow, we intend to publish our findings in open access whenever possible. Finally, the final COS will be published in a major journal (eg, British Medical Journal, Lancet Neurology), given the initiative’s importance and its potential to improve research quality.

We also intend to share our findings with patient associations and through social media. We will disseminate our findings through public engagement activities, based on existing initiatives in their respective countries/institutions, such as European Researchers' Night, alongside collaborations with private insurance companies (ie, AXA and MMA).

The COS will emerge from a consensus process designed to improve the quality and relevance of research evidence in neurocritical care RCTs involving patients with TBI. We expect that the COS will be used only in the RCTs but may also be valuable in other types of research, such as observational studies (eg, registries) and as quality indicators for clinical care.

### Conclusion

This project aims to improve the integrity, transparency, usability, and impact of research related to patients with moderate-to-severe TBI. It will ensure that outcomes relevant to all stakeholders are consistently reported in trials, thereby minimizing outcome reporting bias. Ultimately, this will protect patients from potential harm, enable patients and clinicians to make informed treatment decisions, and allow researchers and policy makers to maximize the public value of research.

## Appendix

Multimedia Appendix 1 Topic guide for patients with traumatic brain injury (TBI) used in Step 2 of developing a core outcome set (COS) in neurocritical care.

Multimedia Appendix 2 Caregivers’ topic guide used for Step 2 of developing a core outcome set (COS) in neurocritical care for patient with traumatic brain injury (TBI).

## Abbreviations

COMET: Core Outcome Measures in Effectiveness Trials
COREQ: Consolidated Criteria for Reporting Qualitative Health Research
COS: core outcome set
GRADE: Grading of Recommendations, Assessment, Development, and Evaluation
IPA: Interpretative Phenomenological Analysis
IRB: institutional review board
OMERACT: Outcome Measures in Rheumatology
PROM: Patient- Reported Outcome Measure
RCT: randomized controlled trial
TBI: traumatic brain injury

## Data Availability

Publications will be made accessible as much as possible (eg, the Step 1 systematic review), depending on available funding. Full data may be available provided upon adequate request to the corresponding author.
