# Supplementary material for: Standardized Outcomes for Randomized Controlled Trials Targeting Early Interventions in Patients With Moderate-to-Severe Traumatic Brain Injury: Protocol for the Development of a Core Outcome Set
Source: JMIR Res Protoc. 2025 Jan 9;14:e54525. doi: 10.2196/54525 (PMC11757975; doi:10.2196/54525)
Supplement: Multimedia Appendix 1 [file resprot_v14i1e54525_app1.docx]

# Appendix 1: Patient with TBI, topic guide

## Objectives and Areas to Explore

**Common Aspects for Both the Patient and the Caregiver:**

- Examine the differences in perspectives between the patient and the caregiver regarding the impact of functional losses.
- Identify the (re)prioritizations in their areas of interest. What is important to them?

**Patient-perspectives:**

- Distinguish between reconstructed memories and the patient’s authentic memories.
- Assess the patient’s awareness of his or her losses. Is this awareness burdensome, and how does he or she adapt?
- Explore the foundations of his or her current life.
- Evaluate the patient's capacity to participate in a semi-structured interview for patients with traumatic brain injury (TBI).

**Before interview and Overview**

- Outline the objectives of the research.
- Emphasize the freedom to participate.
- Explain the necessity of recording for research purposes.
- Detail the data processing methods.
- Highlight the intention to disseminate findings through an article and presentations at conferences.

## Interview Procedure

**Introduction:**

- Questions to be asked are highlighted in yellow.
- Probing questions are underlined.
- Areas to identify in the discourse are italicized for exploration if necessary.
- Advice is indicated in red.
- **Basic Rules:** Avoid closed questions; encourage the participant to discuss situations from his or her own experiences. Respect silences as they may be meaningful.
- Reformulate questions if there are difficulties in understanding.
- Be aware for signs of fatigue.

**Initiate Recording:**

**Introduction:**

- Could you please introduce yourself to begin?

Explore the patient’s living situation, family circumstances, identification of a natural caregiver, educational background, professional history, and areas of interest. Do not prompt responses, as these will be revisited later.

**Theme 1: The Patient’s Experience of Traumatic Brain Injury**

- Can you describe how your accident occurred?
  - Following your accident, do you remember regaining consciousness? How did that happen?
  - Were you hospitalized in the intensive care unit? Can you describe that experience?
  - Where did you go after the intensive care unit?
  - How did your memories return to you? Are these truly your memories?

Investigate the patient’s memories, perceived sequelae, recollections of life before the injury, and the significance of these memories in his or her current experience. Identify how his or her memories resurfaced.

**Theme 2: Current Health Status**

- How are you feeling today?
  - What are your current priorities? What do you enjoy doing?
  - How does your caregiver assist you today?
  - How do you spend your weekends?
  - If you were to envision your life in a year, what could you share about the year ahead when we meet again?

Explore future perspectives, relationships with the caregiver, notions of autonomy, and sources of pleasure, displeasure, comfort, and discomfort, including romantic relationships.

**Theme 3: Patient Priorities**

- Can you summarize what is important to you in your life?

Understand the patient's priorities from his or her perspective, encouraging a reflective approach if the patient is able.
