# Supplementary material for: Standardized Outcomes for Randomized Controlled Trials Targeting Early Interventions in Patients With Moderate-to-Severe Traumatic Brain Injury: Protocol for the Development of a Core Outcome Set
Source: JMIR Res Protoc. 2025 Jan 9;14:e54525. doi: 10.2196/54525 (PMC11757975; doi:10.2196/54525)
Supplement: Multimedia Appendix 2 [file resprot_v14i1e54525_app2.docx]

# Appendix 2: Caregivers’ topic guide

## Objectives and Areas to Explore

#### Common Aspects for the Patient and the Caregiver:

- Examine the differences in perspectives between the patient and the caregiver regarding the impact of functional losses.
- Identify the (re)prioritizations in their areas of interest. What is important to them?

#### Caregiver-perspectives:

- Assess caregiver burden.
- Analyze the discrepancies between the narratives of the patient and the caregiver.
- Evaluate how the caregiver has reprioritized the patient’s areas of interest.

**Objective 1: Patient Experience**

- Explore the patient’s quality of life (QoL) through their lived experience, focusing on changes over time. This includes patients with recently acquired disabilities (RAD) lasting less than two years, and understanding how the RAD is experienced by the caregiver.

**Objective 2: Caregiver Experience**

- Investigate the caregiver’s QoL by exploring their lived experience.

**Before interview and Overview**

- Outline the objectives of the research.
- Emphasize the freedom to participate.
- Explain the necessity of recording for the research.
- Detail data processing methods.
- Highlight the intention to disseminate findings through an article and presentations at conferences.

## Interview Procedure

**Introduction:**

- Questions to be asked are highlighted in yellow.
- Probing questions are underlined.
- Areas to identify in the discourse are italicized for exploration if necessary.
- Advice is indicated in red.
- **Basic Rules:** Avoid closed questions; encourage the participant to discuss situations from their own experiences. Respect silences as they may be meaningful.

**Initiate Recording**

**Introduction:**

- Could you please introduce yourself to begin?

Explore the patient’s living situation, family circumstances, educational background, professional history, and areas of interest without prompting responses, as these will be revisited later.

**Theme 1: The Caregiver’s Experience of the Traumatic Brain Injury**

- Can you describe how the accident occurred? What does the accident signify for you?
  - How was your experience in the intensive care unit?
  - Where did the patient go after leaving the intensive care unit?

Focus on the Caregiver if needed.

Discuss their memories of the event, the significance of these memories in their current experience, and recollections related to care.

**Theme 2: The Patient’s Experience Before the Incident**

- How would you describe your loved one before the accident?
  - How did they interact with those around them?
  - What were their aspirations for the future?

Investigate the patient’s personality and the discrepancies between the caregiver’s narrative and the patient’s narrative, including areas of interest and social networks.

**Theme 3: Current Health Status of the Patient**

- How is the patient doing today?
  - What assistance do they require?
  - Do they need help with daily activities?
  - If you were to envision the patient’s life in a year, what could you share about their life over the next year?

Examine future perspectives, relationships with the patient, notions of autonomy, and sources of pleasure, displeasure, comfort, and discomfort, including romantic relationships.

**Theme 4: The Caregiver’s Daily Life with the Patient and the Impact of TBI**

- How does your daily life unfold?
  - In what ways has the accident changed your life?
  - How does your family cope with the situation?

Investigate caregiver burden, the relationship with the patient, and family and social dynamics.

**Theme 5: Caregiver Priorities**

- Can you summarize what is important in your life during this interview?
  - Do you have any solutions or suggestions to propose?
